# Socio-Demographic Factors and Body Image Perception Are Associated with BMI-For-Age among Children Living in Welfare Homes in Selangor, Malaysia

**DOI:** 10.3390/nu11010142

**Published:** 2019-01-11

**Authors:** Nur Nabilla A Rahim, Yit Siew Chin, Norhasmah Sulaiman

**Affiliations:** 1 Department of Nutrition and Dietetics, Faculty of Medicine and Health Sciences, Universiti Putra Malaysia, Malaysia; nabilla022017@gmail.com (N.N.A.R.); norhasmah@upm.edu.my (N.S.); 2 Research Centre of Excellence, Nutrition and Non-Communicable Diseases, Faculty of Medicine and Health Sciences, Universiti Putra Malaysia, 43400 UPM Serdang, Selangor, Malaysia

**Keywords:** children, welfare home, body image, obesity, BMI-for-age

## Abstract

Considering the double burden of malnutrition in Malaysia, data on malnourished children living in welfare homes are limited. This study aimed to determine the body weight status of children living in welfare homes and its associated factors. A total of 307 children aged 7–17 years old living in 15 selected welfare homes completed a standardized questionnaire, and their body weight and height were measured by trained researchers. There were 54.4% orphans, 23.8% abandoned children, and 21.8% children from problematic families. There were 51.5% boys and 48.5% girls; 52.4% were Malays, followed by 31.3% Indians, 12.7% Chinese, and 3.6% from other ethnic groups. The prevalence of overweight and obesity (23.1%) was higher than the prevalence of thinness (8.5%). In bivariate analyses, socio-demographic factors of age (*p* = 0.003), sex (*p* = 0.0001), ethnicity (*p* = 0.001), and welfare home enrollment status (*p* = 0.003), and psychological factors of self-esteem (*p* = 0.003), body shape dissatisfaction (*p* = 0.0001), and underestimation of body weight status (*p* = 0.002), were significantly associated with body mass index (BMI)-for-age. In the multiple linear regression analysis, children who were either Malays (β = 0.492) or Chinese (β = 0.678), with a status of being abandoned (β = 0.409), with body shape dissatisfaction (β = 0.457), and underestimated body weight status (β = 0.628) significantly explained 39.7% of the variances in higher BMI-for-age (F = 39.550; *p* < 0.05). Besides socio-demographic background, the current findings emphasized the importance of incorporating body image perception in an obesity prevention intervention program in welfare homes.

## 1. Introduction

While obesity has become a major nutritional problem worldwide, childhood obesity is a subject matter of priority as it determines obesity in adulthood and increases the risk of adult morbidity and mortality [1]. In a society where most adults, as well as children and adolescents, are attempting to lose weight, it is foreseeable that weight concerns and poor body image perception (body shape dissatisfaction and misperception of body weight status) are common. 

Body image is a multidimensional construct encompassing how an individual perceive, think, feel, and act toward one’s own body [2], and lies on a continuum from accurate and positive body perceptions to inaccurate and negative body perceptions [2,3]. With the growing concerns of body weight status among children, body dissatisfaction and body image misperception may affect their eating behaviors when they want to achieve an ideal body image and obtain a sense of control of their bodies, which can eventually cause clinical eating disorders and unhealthy body weight status [4,5]. Individuals who have poor body image perception have a low probability of taking part in healthy weight management behaviors; rather, they are more likely to adopt behaviors that could place them at risk of malnutrition and poor health status [6,7].

Body image perception as an associated factor with obesity has been widely discussed in the literature [8,9,10,11]. Previous local studies revealed that body shape dissatisfaction and misperception of body weight status were associated with unhealthy body weight status of Malaysian adolescents [12,13]. However, to the best of our knowledge, no prior studies in the related literature have assessed body shape dissatisfaction and misperception of body weight status among children living in welfare home setting. It is crucial to assess body image perception of these children in preventing unrealistic weight goals and unhealthy weight control behaviors, which may affect their growth and development.

A welfare home is considered to provide a foster family for children in need of care, such as orphans, abandoned children, and children from problematic families. In Malaysia, welfare homes are registered under the Social Welfare Department of Malaysia. The legal caregivers in welfare homes protect the children and provide them with basic necessities such as shelter, food, education, clothing, and school equipment. While a double burden of malnutrition is reported in Malaysia [14], data on malnourished children living in welfare homes are very limited. To the best of our knowledge, there are two local studies that reported about malnutrition among children living in welfare homes [15,16]. In 2008, Chee et al. [15] found that the prevalence of undernutrition (21.0%) was about five times higher than the prevalence of overnutrition (4.0%) among 73 children aged six to 17 years old in Kuala Lumpur. However, in 2015, Mohd Dzulkhairi et al. [16] reported that the prevalence of overnutrition (32.1%) was higher than that of undernutrition (6.2%) among 128 children below 18 years old in Selangor and Melaka. There is a shift in the malnutrition trend among children living in welfare homes from undernutrition to overnutrition within the last seven years. In the present study, malnutrition is determined based on body weight status of the children, assessed using *z*-score of body mass index (BMI)-for-age [14,15,16]. 

Children growing up in out-of-home care, such as those living in welfare homes, may have higher risks of adverse physical, emotional, and behavioral-related outcomes, which may further elevate risks of unstable life-course trajectories in later adulthood life [17,18,19,20]. As compared to children in the general population, children living in welfare homes have higher rates of chronic health conditions (CHCs) [21], including mental retardation and malnutrition. Similarly, children in the formal foster care were found to have high rates of health and mental health problems [22,23]. While depressive disorders refer to the presence of depression symptoms [24], Turney and Wilderman [22] reported that children placed in foster care have a greater likelihood of having depressive disorders than their counterparts. In addition, a previous study by Gürsoy et al. [25] has shown that children living in orphanages had low self-esteem scores, in which self-esteem reflects a person’s overall evaluation or appraisal of one’s own worth [26]. In spite of that, the potential association of self-esteem and depressive disorders with body weight status in the welfare home setting has not been explored further.

Previous reports by Chee et al. [15] and Mohd Dzulkhairi et al. [16] were limited to the study of dietary factors in relation to body weight status among girls [15], and knowledge, attitude, and practice with respect to nutritional status [16], respectively. Nevertheless, the inclusion of socio-demographic and psychological factors in the current study may facilitate the development of strategies for the prevention of disease related to nutritional status among children living in welfare homes. Therefore, this study aims to determine the body weight status among children living in welfare homes, and its association with socio-demographic and psychological factors. 

## 2. Materials and Methods 

### 2.1. Study Design and Respondents

This cross-sectional study was carried out in the Selangor state of Malaysia. Respondents of the present study were children living in welfare homes in Selangor, where there were three main categories of children, namely orphans, abandoned children, and children from problematic families. Children from problematic families included those with divorced parents, who lived in poverty, and were formerly abused by members of the family. 

Based on the probability proportionate sampling, 524 children from 15 selected welfare homes were invited to participate in the current study. A total of 307 children who fulfilled the inclusion criteria of the study consented to participate in the study. The inclusion criteria of the children for this study were Malaysian, registered children in welfare homes and enrolled school-going children aged from 7 to 17 years old. Meanwhile, this study excluded children with disabilities and children who had physical and mental illnesses based on their health records. 

The present study was conducted in accordance with the Declaration of Helsinki. Ethical approval was attained from the Ethics Committee for Research Involving Human Subjects, Universiti Putra Malaysia (UPM/TNCPI/RMC/1.4.18.1 (JKEUPM)/F2). Permissions to conduct this study were obtained from the Department of Social Welfare, Malaysia, and all selected welfare homes. Researchers explained about the study protocol to the children and their caregivers at the selected welfare homes, and informed consents were obtained from both the children and their caregivers. They were informed about their rights to voluntarily withdraw from the study. After getting informed consents from the caregivers and children, anthropometric measurements were taken by trained researchers. Respondents aged 10 years and above were requested to self-administer the questionnaire, whereas respondents aged below 10 years were interviewed by the researchers. 

### 2.2. Study Measurements

#### 2.2.1. Anthropometric Measurements

Body weight and height of respondents were measured with them in light clothing and barefooted using TANITA digital weighing scale Model HD-382 (TANITA Corporation, Japan) to the nearest 0.1 kg, and a portable SECA stadiometer Model 213 (SECA, Germany) to the nearest 0.1 cm, respectively. BMI-for-age (*z*-score) was determined by using the WHO Anthro- version 1.0.3 software [27]. Body weight status was classified using the WHO Growth Reference for 5-19 years [28]. 

#### 2.2.2. Socio-Demographic Factors

Caregivers of the welfare homes were interviewed by using a socio-demographic questionnaire requesting information about the date of birth (age), sex, ethnicity, and enrollment status of the respondents. 

#### 2.2.3. Psychological Factors

##### Self-Esteem

Self-esteem was measured using the 10-item Rosenberg Self-Esteem Scale [26]. For items 1, 2, 4, 6 and 7, respondents had to give a score, with 1 = strongly agree, 2 = agree, 3 = disagree, and 4 = strongly disagree. Meanwhile, for items 3, 5, 8, 9 and 10, respondents had to give a score, with 1 = strongly disagree, 2 = disagree, 3 = agree, and 4 = strongly agree. The scores of all items were summed up to obtain the total self-esteem score, which ranged from 10 to 40. The categorization of the level of self-esteem was based on a previous research among children who lived in orphanages in Sharkia governorate [29], whereby a self-esteem score of below 15 signified low self-esteem. The internal consistency of the scale in this study was 0.53.

##### Depressive Disorders

The respondents’ depressive disorders were assessed using the Children Depression Index [30], which is a standardized self-report developed for children and adolescents aged 6–17 years old. The questionnaire used in this study was an adapted version whereby a statement on “I want to kill myself” was removed, as it did not suit the Malaysian and welfare home context. There were 26 items, on a scale from zero (not a problem) to two (severe). The scores for all items were summed up to obtain a total score, which ranged from 0 to 52. A score of 19 and above indicated that the respondent had a higher likelihood of having depressive disorders [31]. The internal consistency of the scale in this study was 0.83.

##### Perception of Body Shape

A seven-figure Collins’ body image silhouette [32] was used to assess the perception of body shape for children aged 7–12 years old, while a nine-figure Contour Drawing Rating Scale [33] was used to assess the perception of body shape for children aged 13–17 years old. Respondents were required to select the figure that resembled their actual body figure and the ideal body figure, respectively. Subtraction of the numeric values that corresponded to “actual” and “ideal” figures were used to calculate the body shape discrepancy score. The score indicated the degree of body shape dissatisfaction. A positive body shape discrepancy score signified the preference for a thinner body, while a negative score signified the preference for a bigger body. 

##### Perception of Body Weight Status

Respondents were asked about their perception of body weight status [34]. The response options were “very thin” (1), “thin” (2), “normal” (3), “overweight” (4), and “obesity” (5). Then, the actual body weight status was compared with the perceived body weight status. Respondents were classified as correct estimators (perception equal to actual body weight status), under-estimators (perception is smaller than actual body weight status), and over-estimators (perception is bigger than actual body weight status). 

### 2.3. Statistical Analyses

Statistical analyses were performed using IBM SPSS Statistics 21 software (version 21.0; SPSS Inc., Chicago, IL, USA), with a significance level set at *p* < 0.05. Frequency, percentage, mean, and standard deviation were used to describe the respondents’ characteristics. The normality of the data was assessed by using the skewness test of normality. All continuous data of the present study were normally distributed, whereby a normal distribution was considered by the value of skewness within the range of ± 2.0 [35]. Statistical tests including the Pearson’s product–moment correlation, independent samples *t*-test, one-way ANOVA, and chi-squared test were used in bivariate analyses, while a multiple linear regression (stepwise method) was performed to determine the factors associated with BMI-for-age. 

## 3. Results

### 3.1. Body Weight Status of the Respondents

**Figure 1:**
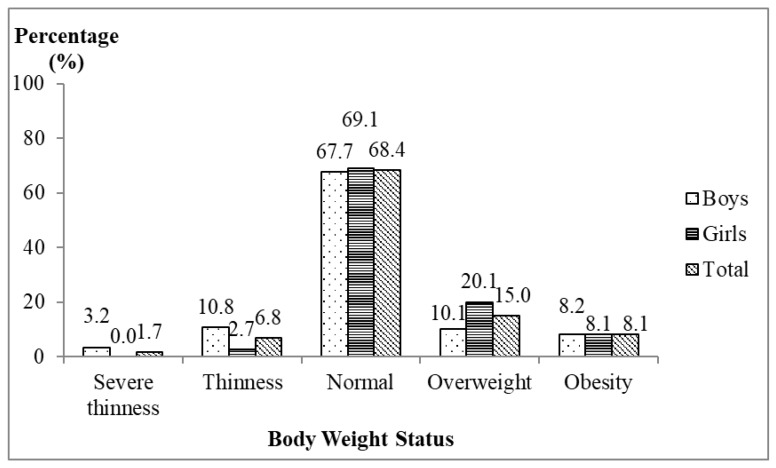
Distribution of respondents according to body weight status (*N* = 307).

The mean body weight and height of the respondents were 43.1 ± 15.6 kg and 147.0 ± 14.0 cm, respectively. The mean BMI was 19.4 ± 4.7 kg/m^2^ and the mean *z*-score of BMI-for-age was −0.06 ± 1.43. The present study found that about one-third of the respondents were facing malnutrition problems. Figure 1 shows that the prevalence of overweight and obesity were 15.0% and 8.1%, respectively, while the prevalence of severe thinness and thinness were 1.7% and 6.8%, respectively.

### 3.2. Socio-Demographic Factors of the Respondents

The study consisted of a total of 307 respondents, with 51.5% boys and 48.5% girls (mean age of 13.0 ± 2.7 years old). About half of them (52.4%) were Malays, followed by 31.3% Indians, 12.7% Chinese, and 3.6% from other ethnic groups. More than half of the respondents living in the welfare homes were orphans (54.4%), followed by 23.8% abandoned children, and 21.8% children from problematic families (those with divorced parents, from poor families, or who had been abused).

### 3.3. Psychological Factors of the Respondents

Table 1 demonstrates the distribution of respondents according to psychological factors. The prevalence of low self-esteem was 16.3%, with a mean score of self-esteem was 18.2 ± 3.7. Meanwhile, the prevalence of high likelihood of depressive disorders was 38.4%, with a mean score of 16.5 ± 7.7. As for body image perception, the study found that the prevalence of body shape dissatisfaction was 73.0%, with a mean score of 0.1 ± 1.7. On the other hand, 37.8% of the respondents had reported to have underestimated their actual body weight status. 

### 3.4. Associations between Socio-Demographic and Psychological Factors with BMI-for-Age

Table 2 shows the bivariate analyses of socio-demographic factors and BMI-for-age. Age was positively correlated with BMI-for-age of the respondents (*r* = 0.169; *p* < 0.05). Overall, girls had significantly higher BMI-for-age compared to boys (*t* = −2.833; *p* < 0.05). Across ethnicities, Malays and Chinese respondents had significantly higher mean BMI-for-age compared to Indians and other ethnic groups (F = 5.358; *p* <0.05). By enrollment status, children being abandoned had the highest mean BMI-for-age compared to orphans and children from problematic families (F = 5.926; *p* < 0.05).

Table 3 shows the bivariate analyses of psychological factors and BMI-for-age. Self-esteem score had inverse correlation with BMI-for-age (*r* = −0.112; *p* < 0.05). However, there was no correlation between the likelihood of depressive disorders and BMI-for-age (*r* = 0.092; *p* > 0.05). Body shape dissatisfaction was significantly correlated with BMI-for-age (*r* = 0.551; *p* < 0.05). In terms of perception of body weight status, respondents who underestimated their body weight status had the highest mean BMI-for-age compared to other categories (F = 6.523, *p* < 0.05). 

Further, multiple linear stepwise regression was conducted to determine the factors contributing to BMI-for-age (*z*-score). As shown in Table 4, respondents who were abandoned, either Malay or Chinese, were dissatisfied with their body shape, and underestimated their body weight status had a higher BMI-for-age (*z*-score). The strongest factor of the BMI-for-age model was body shape dissatisfaction (∆R^2^ = 30.4%), followed by underestimation of body weight status (∆R^2^ = 4.8%), Malay (∆R^2^ = 1.7%) or Chinese (∆R^2^ = 1.6%) ethnic group, and abandoned status (∆R^2^ = 1.2%), which explained a total of 39.7% of the variances in BMI-for-age of the respondents (F = 39.550; *p* < 0.05).

## 4. Discussion

The body weight status of children living in welfare homes in Selangor was unsatisfactory. Dual forms of malnutrition existed among children living in welfare homes, whereby the prevalence of overweight and obesity (23.1%) was about three times higher than the prevalence of thinness and severe thinness (8.5%). When compared to the recent report of the Adolescent Health Survey 2017 among school students aged 10 to 17 years old by Malaysia country and Selangor state [36], children living in the welfare homes generally had a lower overweight and obesity prevalence. The prevalence of overweight and obesity was 30.4% according to the national prevalence and 33.0% according to the Selangor state report, respectively [36]. On the other hand, more respondents were found thin in the welfare homes compared to the national survey. There were 6.6% and 6.7% of them were thin based on the national prevalence and the Selangor state prevalence, respectively [36]. 

In contrast to the current findings, previous studies of welfare homes in other countries such as Bangladesh, Nigeria, Ghana, and Sri Lanka reported that the prevalence of underweight was higher than that of overweight [37,38,39,40]. Nonetheless, the current findings are in agreement with a study among children living in welfare homes in Southeast Nigeria, wherein the prevalence of overweight (30.0%) was higher than the prevalence of underweight (2.2%) [41]. Our findings did not correspond to a local study by Chee et al. [15] in 2008, whereby they found that the undernutrition issue was more prevalent than overnutrition. However, a direct comparison of findings cannot be made as the present results used the WHO Growth Reference for those aged 5–19 years [28], while Chee et al. [15] classified children based on the Centers for Disease Control and Prevention (CDC) BMI-for-age Growth Chart [42]. As reported by Mohd Dzulkhairi et al. [16] in 2015, the trend of malnutrition among children living in welfare homes has changed over the last decade since Chee et al. [15] conducted the study in 2008. Our findings supported the findings of Mohd Dzulkhairi et al. [16] who reported that the prevalence of overweight and obesity (32.1%) was higher than that of thinness (6.2%), using the WHO (2007) classification. Based on our observation and informal communication with the caregivers, the current overweight and obesity problems among children living in the welfare homes may be due to the surplus of energy-dense foods such as pizza, fried chicken, hamburgers, fries, carbonated drinks, and ice-cream received from donors, which might lead to overeating. Possibly, children might be afraid of being hungry, and thus overeat whatever foods are given by their caregivers. Furthermore, children living in the welfare homes have always been given the opportunity to eat out, especially on weekends, and they usually go to western fast food outlets. An in-depth study about the eating behaviors and dietary intakes of children living in the welfare homes is needed.

The present study found that self-esteem was inversely associated with BMI-for-age in the bivariate analysis. Consistent with previous studies [43,44], low self-esteem was associated with the risk of overweight and obesity. The plausible explanation that low self-esteem is linked to overweight and obesity among the respondents was because children who have low self-esteem manifest their loneliness through disturbed eating habits and negative emotions related to eating during their stay in welfare homes. Nonetheless, there was no further significant association found between self-esteem and BMI-for-age in the multivariate analysis of the current findings. While about one-third of the children living in welfare homes had a high likelihood of having depressive disorders, there was no significant association between depressive disorders and BMI-for-age in the present study. Contradictory to a study by Luppino et al. [45], there was a reciprocal relationship between mental health and body weight status in which they reported that depression may be both a factor and an impact of obesity. On the other hand, the current findings are aligned with previous studies that focused on children in the general population, which reported no association between depression and body weight status [46]. In addition, a meta-analysis study revealed no significant association between body weight status and depression in the general population [47]. Although self-esteem and depressive disorders did not significantly contribute towards BMI-for-age of the children living in the welfare homes, both factors are still crucial to be addressed among children living in the welfare homes in promoting psychological stability and positive social activity in their later years of life.

Furthermore, the current study found that respondents with high BMI-for-age were those who were dissatisfied with their body shape, underestimated their body weight status, were Malays or Chinese, and had been abandoned. These factors explained about 40% of the variances in the BMI-for-age model. The current regression model was compared with other models on BMI-for-age in the Selangor state of Malaysia. It should be noted that our model was compared to the general population of Malaysia due to limited studies targeting respondents living in the welfare homes as their study respondents. For instance, a previous study in the Kajang district, Selangor, indicated that physical activity, body image, and energy intake explained 36.9% of the variances in BMI-for-age of adolescents aged 13 to 15 years old [48]. Physical activity was not included in the present study, yet our body image indicators, namely body shape dissatisfaction and body weight status underestimation, were consistent with the factors of negative body image found in their model [40]. Another BMI-for-age model of early adolescents aged 10 to 11 years old in Selangor showed that energy expenditure per kilogram body weight, being male, and the BMI of the mothers explained 66.7% of the variances in high BMI-for-age model [49]. In contrast to the model, our study found that sex did not contribute towards the high prevalence of overweight and obesity, while energy expenditure and maternal BMI were not assessed.

The present study also showed that Malay or Chinese respondents had higher BMI-for-age compared to other ethnicities. A previous study among Malaysian primary school children reported that the prevalence of overweight was higher among Chinese students (23.0%) compared to Indians (16.0%) and Malays (14.8%) [50]. Additionally, the prevalence of obesity was higher in Malays (7.6%), followed by Indians (5.1%) and Chinese (1.6%) [49]. Further studies may need to explore the underlying differences in ethnic groups that relate to the development of overweight and obesity in children. For instance, Zhang and Wang [51] suggested that Chinese people perceived having a large body size or being overweight and obese as a sign of wealth and success in their culture in China.

By enrollment status of the welfare homes, respondents with the enrollment by abandoned status had the highest BMI-for-age, followed by orphans, and children from problematic families. The possible explanation for these findings may be due to the immediate environment experienced by children who were abandoned from a young age (for example as newborns) when entering the welfare homes compared to other children. The immediate environment here refers to the welfare home as the first home of children being abandoned, which may be an obesogenic environment that encourages children to become overweight and obese from a very young age. On the other hand, most of the orphans and children from problematic families lived in their parents’ home as their first home, which may indicate different early environment exposures as compared to abandoned children. Future research may need to consider the environment exposure prior to the enrollment when focusing on children living in welfare homes.

To the best of the authors’ knowledge, no previous study in the related literature has assessed the perception of body image in the welfare home setting. However, the present findings support a study among children of general population from Nazrat et al. [52], in which girls desired for a smaller body size than boys. Likewise, Ozmen et al. [53] indicated that boys desired a larger body size. Furthermore, the association between body shape dissatisfaction and obesity was found in several studies in Malaysia [48,54,55]. Meanwhile, the present findings showed that there was an association between respondents under-estimating their body weight status and overweight and obese status. This is consistent with a study among adolescents of the general population aged 10 to 17 years in Malaysia, where the authors found that 12.2% of the overweight and obese respondents had under-estimated their actual body weight status [13]. Neumark-Sztainer et al. [56] highlighted that the association between poor body image (body shape dissatisfaction and underestimation of body weight status) and obesity was due to unhealthy weight control behaviors that put them at risk of weight gain. Further studies may focus on determining the types of unhealthy weight control behaviors among children. 

There were several limitations in the current study. This cross-sectional study does not yield the evidence on the cause and effect of socio-demographic and psychological factors on BMI-for-age. However, the present study adds important information to the literature regarding the association of socio-demographic and psychological factors with body weight status among children living in welfare homes. Immediate action must be initiated to prevent excess weight gain and to treat those children who are already in the high BMI-for-age category.

## 5. Conclusions

The present study found that dual forms of malnutrition existed among children living in welfare homes. About one in four of the children were facing overweight and obesity problems, whereas about one in ten of them were thin and severely thin. Poor body image perception, being Malays or Chinese, and having been abandoned were associated with higher BMI-for-age among children living in welfare homes in Selangor. These findings suggest the need to have a regular assessment of body weight status among children, and preventive actions should be taken by the welfare homes’ related agencies and donors. Additionally, an obesity intervention program that incorporates body image perception may improve the children’s body weight status in future.

## Figures and Tables

**Table 1:** Distribution of respondents according to psychological factors (*N* = 307).

Psychological Factors	Boys (*n* = 158)		Girl (*n* = 149)		Total (*N* = 307)		*t/χ^2^*	*p*-Value
	Mean ± SD	*n* (%)	Mean ± SD	*n* (%)	Mean ± SD	*n* (%)		
**Self-esteem**	18.7 ± 3.9		17.6 ± 3.4		18.2 ± 3.7		2.565 ^a^	0.011 *
Low		25 (15.8)		25 (16.8)		50 (16.3)		
Normal		133 (84.2)		124 (83.2)		257 (83.7)		
**Depressive disorders**	16.0 ± 7.7		16.9 ± 7.7		16.5 ± 7.7		−1.045 ^a^	0.297
No likelihood of a depressive disorder		105 (66.5)		84 (56.4)		189 (61.6)		
High likelihood of a depressive disorder		53 (33.5)		65 (43.6)		118 (38.4)		
**Perception of body shape**	−0.5 ± 1.5		0.8 ± 1.7		0.1 ± 1.7		−7.185 ^a^	0.0001 *
Satisfied		50 (31.6)		33 (22.1)		83 (27.0)		
Dissatisfied		108 (68.4)		116 (77.9)		224 (73.0)		
**Perception of body weight status**							2.242 ^b^	0.326
Correct-estimator		79 (50.0)		86 (57.7)		165 (53.7)		
Over-estimator		13 (8.2)		13 (8.7)		26 (8.5)		
Under-estimator		66 (41.8)		50 (33.6)		116 (37.8)		

*n*, number of respondents; *N*, the sample size of this study; ^a^ refers to *t*, a statistic that compared whether sexes had different means through the independent samples *t*-test; ^b^ refers to *χ*^2^, a statistic used for testing associations between categorical variables through chi-squared test; * Statistical significance at *p* < 0.05.

**Table 2:** Association between socio-demographic factors and BMI-for-age (*z*-score) (*N* = 307).

Socio-Demographic Factors	BMI-for-Age (*z*-Score)	*r*/*t*/F-Value	*p*-Value
	Mean ± SD		
**Age of respondents**		0.169 ^b^	0.003 *
**Sex**		−2.833 ^c^	0.0001 *
Boys	−0.36 ± 1.54		
Girls	0.26 ± 1.23		
**Ethnicity**		5.358 ^d^	0.001 *
Malay	0.01 ± 1.37		
Chinese	0.60 ± 1.26		
Indian	−0.43 ± 1.47		
Others ^a^	−0.19 ± 1.61		
**Enrollment status**		5.926 ^d^	0.003 *
Orphan	−0.12 ± 1.51		
Abandoned	0.39 ± 1.25		
Problematic family	−0.41 ± 1.30		

*N*, sample size of this study was 307; ^a^ Others refer to aborigines and ethnics from Sabah and Sarawak; BMI, body mass index; SD, standard deviation; ^b^ refers to *r*, the correlation coefficient that measured the strength and direction of a linear relationship between two variables through Pearson’s correlation analysis; ^c^ refers to *t*, a statistic that compared whether sexes had different means through the independent samples *t*-test; ^d^ refers to F, a statistic that compared differences between means of more than two groups through one-way ANOVA; *Statistical significance at *p* < 0.05.

**Table 3:** Association between psychological factors and BMI-for-age (*z*-score) (*N* = 307).

Psychological Factors	BMI-for-Age (*z*-Score)	*r*/F-Value	*p*-Value
	Mean ± SD		
Self-esteem		−0.112 ^a^	0.003 *
Likelihood of depressive disorders		0.092 ^a^	0.109
Body shape dissatisfaction		0.551 ^a^	0.0001 *
Perception of body weight status		6.523 ^b^	0.002 *
Under-estimator	0.25 ± 1.60		
Correct-estimator	−0.16 ± 1.22		
Over-estimator	−0.77 ± 1.56		

*N*, the sample size of this study, was 307; BMI, body mass index; SD, standard deviation; ^a^ refers to *r*, the correlation coefficient that measured the strength and direction of a linear relationship between two variables through Pearson’s correlation analysis; ^b^ refers to F, statistic that compared differences between means of more than two groups through one-way ANOVA; *Statistical significance at *p* < 0.05.

**Table 4:** Multiple linear stepwise regression of BMI-for-age (*z*-score) (*N* = 307).

Variables	Unstandardized Coefficients		Standardized Coefficients	*t*	∆R^2^	*p*-Value
	β	Standard Error	Beta			
(Constant)	−0.796	0.131		−6.063		0.0001 *
Body shape dissatisfaction	0.457	0.038	0.549	12.126	0.304	0.0001 *
Underestimation of body weight status	0.628	0.134	0.213	4.691	0.048	0.0001 *
Malay	0.492	0.145	0.172	3.396	0.017	0.001 *
Chinese	0.678	0.214	0.158	3.162	0.016	0.002 *
Abandoned status	0.409	0.164	0.122	2.489	0.012	0.013 *

*N*, the sample size of this study, was 307; R, the multiple correlation coefficient, was 0.630; R^2^, the coefficient of determination was 0.397; F, the ratio of the model mean square to the error mean square was 39.550; β, values for the regression equation for predicting the dependent variable from the independent variables, Beta was the standardized coefficient; *t*, a statistic used to check the significance of individual regression coefficients in the regression model; ∆R^2^, incremental increase in the model R^2^ resulting from the addition of a predictor to the regression equation; *Statistical significance at *p* < 0.05.
